# Evolution, Chance, and Aging

**DOI:** 10.3389/fgene.2021.733184

**Published:** 2021-09-09

**Authors:** Stewart Frankel, Blanka Rogina

**Affiliations:** ^1^Biology Department, University of Hartford, West Hartford, CT, United States; ^2^Genetics and Genome Sciences, Institute for Systems Genomics, School of Medicine, University of Connecticut Health Center, Farmington, CT, United States

**Keywords:** aging, evolution, pleiotropy, longevity, senescence, mutation accumulation

## Abstract

Aging has provided fruitful challenges for evolutionary theory, and evolutionary theory has deepened our understanding of aging. A great deal of genetic and molecular data now exists concerning mortality regulation and there is a growing body of knowledge concerning the life histories of diverse species. Assimilating all relevant data into a framework for the evolution of aging promises to significantly advance the field. We propose extensions of some key concepts to provide greater precision when applying these concepts to age-structured contexts. Secondary or byproduct effects of mutations are proposed as an important factor affecting survival patterns, including effects that may operate in small populations subject to genetic drift, widening the possibilities for mutation accumulation and pleiotropy. Molecular and genetic studies have indicated a diverse array of mechanisms that can modify aging and mortality rates, while transcriptome data indicate a high level of tissue and species specificity for genes affected by aging. The diversity of mechanisms and gene effects that can contribute to the pattern of aging in different organisms may mirror the complex evolutionary processes behind aging.

## Introduction

Evolutionary theory has undergone several stages of development – the initial formulation by Darwin and his successors, the advent of population genetics and the evolutionary synthesis, the integration of evolutionary theory with modern developmental biology (Gilbert et al., 1996), and now the stage of assimilating genomic and molecular genetic data. This article expands upon several evolutionary concepts, using insights from contemporary molecular genetic studies, and applies them to the issue of aging.

Aging presents interesting challenges for evolutionary theory. It is part of the life history of many multicellular organisms, but varies from individual to individual in a population (Finch, 1990; Austad, 1997; Herndon et al., 2002; Wilson et al., 2007; Zhang et al., 2016). As a physiological process aging can be complex, since its rate and presentation differ from tissue to tissue. There is even cell-to-cell heterogeneity within tissues (Arrojo e Drigo et al., 2019). Aging is statistically linked to intrinsic mortality, which occurs when a population is under optimal conditions and experiences low levels of extrinsic mortality (such as predation or accidents) (Finch, 1990). Mortality is easy to measure, but the relationship between aging in various tissues and intrinsic mortality is still unclear. Evolutionary theory can treat mortality and aging as phenomena affected by mutations in a population of organisms, without recourse to mechanistic explanations or physiological models. That type of approach places the emphasis on survival and reproduction, or simply fitness, instead of tissue dysfunction. Survival and fecundity are polygenic traits, so that alleles in many genes would be expected to affect each trait. As phenotypes, survival and fecundity are also influenced by environmental factors, adding to their complexity as traits in a population or species. Population and quantitative genetics have provided a highly developed framework of theory and experimental data for understanding aging and its evolution. Mathematical treatments are beyond the scope of this review, which instead aims for a more intuitive approach, but citations are provided in this area.

The discovery of single gene mutations that decrease intrinsic mortality has created a great deal of data about how intrinsic mortality can be modified (Campisi et al., 2019). This is a substantially different approach with regard to understanding the complex genetic basis of aging and survival, which can be complementary to the evolutionary approach or antithetical to it. In such studies longevity increases are viewed as more informative than longevity decreases, since the latter can be caused by processes unrelated to aging (Helfand and Rogina, 2003). A literature search indicates that model organism life span can be increased by mutations in at least 60 *Mus musculus* genes, at least 160 *Drosophila melanogaster* genes, and at least 620 *Caenorhabditis elegans* genes (Tacutu et al., 2018, *Human Aging Genomic Resources*). These numbers partially reflect the constraints for survival studies in each organism. Some of these mutations have also been linked to decreased physiological aging. A systematic RNAi screen in *C. elegans* demonstrated longevity extension with approximately 2% of the tested genes (Lee et al., 2003). A large-scale screen of *Drosophila* P-element insertion mutants determined that 4% of the tested genes increased longevity (Magwire et al., 2010). There seems to be a very diverse array of processes that can affect longevity. Alleles in hundreds of genes could potentially affect intrinsic mortality within each species, while the number may be much larger when comparing a broad cross-section of species. In this respect, the rapidly accelerating generation of whole genome sequences for diverse species and for multiple individuals within populations of the same species should prove to be an important new source of data on genes and alleles correlated with longevity. Candidate genes and gene variants identified by unbiased genomic searches can then provide the basis for further studies in model organisms (Treaster et al., 2021).

This is both a review and a theoretical exploration of some genetic concepts. Given the many areas relevant to aging a comprehensive review is not possible in this format, but reviews are cited that provide detailed and complete analysis of particular topics, while the focus here will be some recent advances.

### Terminology

**FIGURE 1:**
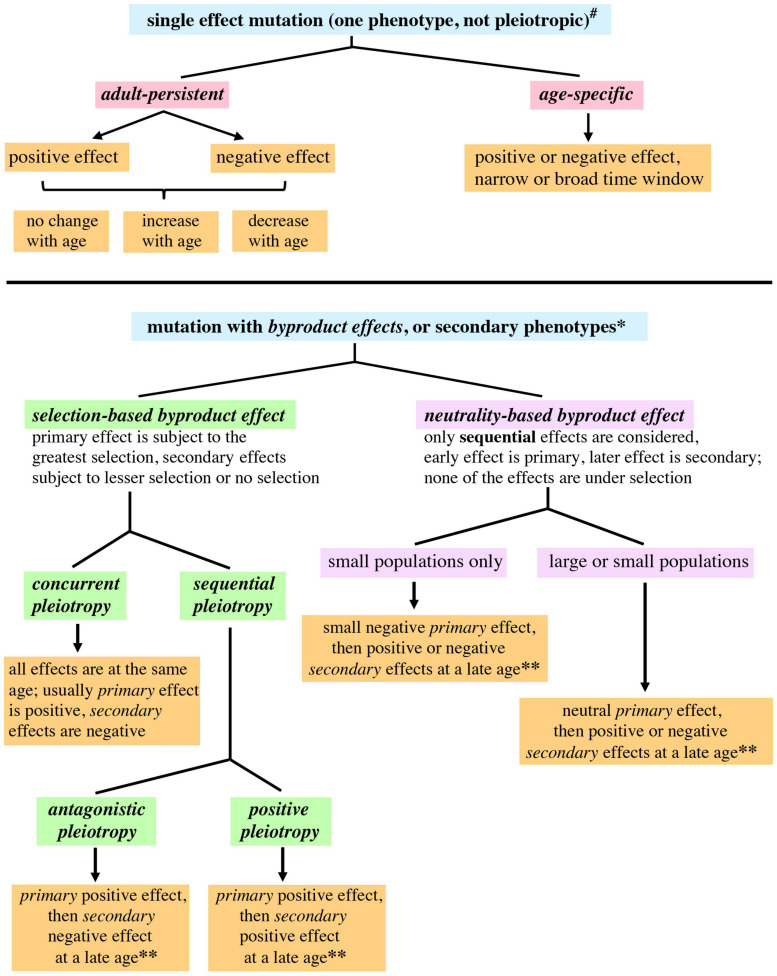
**A proposed expansion of genetic terminology allowing application of the terminology to fitness and survival in age-structured populations**. A mutation that is *pleiotropic* or has *byproduct effects* is defined as having qualitatively different primary and secondary phenotypic effects. *Concurrent* means the multiple effects of the mutation occur in the same life history stage. *Sequential* means the primary and secondary effects of the mutation occur in different life history stages. **^#^** For single effect mutations, “positive” or “negative” refers to phenotypes affecting fitness; phenotypes affecting survival are relevant to the widest range of ages. ^*^Primary effects are described as positive or negative with regard to fitness. For secondary effects occurring at ages that still have appreciable selection, the secondary effects are also described as positive and negative with regard to fitness. Secondary effects occurring at late ages not subject to selection have a different usage of these terms: “positive” and “negative” describe the effect of a mutation upon survival or a phenotype related to vigor (health span). ** Only late onset secondary effects would contribute to aging, since secondary effects occurring at younger ages may be subject to selection.

Instead of discussing an *aging process*, which can be mistaken for a step-by-step process, it might be more helpful to discuss a *pattern of aging*. A *pattern of aging* merely describes aging as a phenomenon. There are several evolutionary considerations that argue against aging as a program or sequential process, analogous to developmental programs (Kirkwood and Austad, 2000; Kirkwood, 2002). Similar considerations also make it unlikely aging is an adaptive physiological response. In order to highlight the age-structured effects of mutations, we propose distinguishing between *adult-persistent* and *age-specific* effects (Figure 1). When discussing mutations that affect late life, *negative effects* would lead to an increased rate of mortality or increased frailty, whereas *positive effects* would lead to a decreased rate of mortality or increased vigor during late life.

Caspari (1952) defined pleiotropy as a single gene affecting multiple traits or characters. However, when the term “pleiotropy” has been adapted to different contexts it has undergone subtle shifts in meaning (Stearns, 2010), leading to usages described in one publication as “molecular gene pleiotropy,” “developmental pleiotropy,” and “selectional pleiotropy” (Paaby and Rockman, 2013). It may be useful to further probe the usage of “pleiotropy,” with the goal of having greater precision when describing age-structured contexts. A pleiotropic mutation can be explicitly defined as involving qualitatively different phenotypic effects, one of which would be primary and the others secondary. A mutation that only has one type of effect can still affect multiple life stages, and vary quantitatively across those stages, but this would not be pleiotropy. In the experimental context of genetic screens, the primary phenotype will be the one that is selected by the researcher, the others will be secondary phenotypes. In an evolutionary context, the primary phenotype will be the one that most strongly affects fitness and is subject to the strongest selection; the secondary phenotypes would have minor effects upon fitness and be under weaker selection, or have no effect upon fitness. Primary and secondary pleiotropic effects can occur at the same stage in an organism’s life history, which can be defined as *concurrent pleiotropy*, or at different stages, which can be defined as *sequential pleiotropy* (see Figure 1). *Antagonistic pleiotropy* and *positive pleiotropy* (discussed below) are *sequential*.

### The Evolution of Traits

There are three possible explanations for the evolution of a trait or phenomenon in a population or species (Masel and Promislow, 2016). (1) It can result from random events or be the cumulative result of random effects. This is usually the null hypothesis. (2) It can result from adaptation mediated by natural selection. (3) It can be a byproduct effect, defined by Masel and Promislow as secondary consequences resulting from selection for a different trait. A byproduct effect will be more generally defined here as the secondary effect of a mutation. The null hypothesis explains a situation as due to chance, without invoking a particular evolutionary mechanism such as adaptation. Natural selection leading to adaptation is an alternative to random changes in a population, and requires a higher level of support. Another alternative that might be as prominent as adaptation is sexual selection, where the selection concerns mate choice. This does not necessarily lead to a higher level of adaptation (Prum, 2017), but it can have a profound effect upon traits in a population. Like natural selection, it requires a higher level of support than the null hypothesis. In the discussion below, either natural or sexual selection will be invoked by the term “selection.”

The mutation accumulation theory proposes the random accumulation of mutations that negatively affect older organisms, leading to increased frailty and mortality (Medawar, 1952; Flatt and Partridge, 2018). Any mutations that affect late life but not early life will not be subject to natural selection, since older individuals represent a small percentage of the total population (a quantitative lessening of selection). Late life will also be a post-reproductive period for most organisms (a cessation of selection). Mutation accumulation is therefore an example of a null hypothesis for answering the question “why does aging happen” (Masel and Promislow, 2016). Two classes of mutations described in Figure 1 would lead to mutation accumulation: age-specific phenotypes with late onset negative effects, and adult-persistent phenotypes with negative effects that increase at later ages. Mutation accumulation presupposes that most spontaneous mutations affecting late life will be negative with regard to survival. However, it has been necessary to postulate the existence of mutations that have persistent positive effects, where the positive effects early in life persist into late life, in order to explain the leveling off of mortality rates at late ages for species that have significant post-reproductive life spans (Charlesworth, 2001; Rauser et al., 2006) (discussed in the next section). New genome sequencing techniques hold the promise of providing an unbiased and comprehensive view of spontaneous genomic mutations (Eyre-Walker and Keightley, 2007). The rate and type of spontaneous mutation could potentially constrain the mutation accumulation process.

Williams (1957) postulated that age-independent extrinsic mortality would be a driver of the evolution of aging and species life span, but that may not be the case if both reproduction and survival are used to model the age distribution of a population and the selection gradient for mortality (Wensink et al., 2017; Moorad et al., 2019). The low number of older individuals in a population could be mainly due to a continual influx of new progeny, and advanced ages would be a small part of the total population even for an immortal species (Wensink et al., 2017). Age-independent extrinsic mortality affects individuals at all ages and modeling suggests that selection gradients are not affected by this type of mortality (Moorad et al., 2019). Additional conditions may be necessary to account for the evolution of aging, such as age-specific mortality or population density effects that differentially affect particular age ranges. A new model predicts that the fixation of beneficial mutations may be more likely in small populations with longer post-reproductive life spans compared to populations with shorter post-reproductive life spans, providing a potential selection pressure for longer life spans (Giaimo and Baudisch, 2015).

The theoretical framework for the evolution of aging has undergone extensive development since the initial formulation of the mutation accumulation theory (Charlesworth, 2000; Kirkwood and Austad, 2000; Rose et al., 2007; Moorad and Promislow, 2008; Moorad et al., 2019; Flatt, 2020). Some of these advancements will be discussed in the next two sections.

### Use of Experimental Evolution to Study Aging

One source of evidence to support evolutionary theories of aging involves comparative studies of life history characteristics exhibited by different species, or different populations within the same species (Matos et al., 2004; Jones et al., 2014; Johnson et al., 2019). Another source of evidence has been experimental evolution, where a population is selected for a particular phenotype in the laboratory (Burke and Rose, 2009; Garland and Rose, 2009; Kawecki et al., 2012). The selection can be short term, spanning 3 (Rose and Charlesworth, 1981) to 50 *Drosophila* generations (Remolina et al., 2012), or long term, spanning either hundreds of *Drosophila* generations (Rose et al., 2002; Burke et al., 2016; Phillips et al., 2016) or many thousands of bacterial generations (Blount et al., 2018; Card et al., 2019). The selection is done under controlled conditions with multiple replicate populations. Experimental evolution has been used with bacteria, unicellular eukaryotes, or metazoans to study many aspects of evolution, testing both the assumptions underlying population genetic models of evolutionary change and the predictions of these models. Studies addressing aging have primarily used several species of fruit fly. The first *Drosophila* studies selected for reproduction late in the life span, using a graded process of successively later reproduction times (Rose and Charlesworth, 1981; Luckinbill et al., 1984; Rose, 1984). These studies were based upon a population genetics model initiated by Hamilton and developed by Charlesworth (reviewed in Rose et al., 2007), where the effect of natural selection upon mortality rates is dependent upon age-specific survival probabilities and age-specific reproductive capacities. Selecting for late reproduction was predicted to result in greater longevity, which was the result obtained independently by several laboratories using *Drosophila melanogaster* (Luckinbill et al., 1984; Rose, 1984; Partridge and Fowler, 1992). Selection for earlier reproduction led to shorter longevity, a result consistent with antagonistic pleiotropy (Rose and Charlesworth, 1981; Luckinbill et al., 1984; Rose, 1984).

Demographic studies of large cohorts in several species revealed that mortality rates increase during most of the adult life span but then flatten or plateau at late ages (Vaupel et al., 1998; Rose et al., 2002; Rauser et al., 2006), with some individuals having an extended period of life after reproduction has ceased. Studies in *Drosophila* indicated that the onset of this plateau was correlated with the age at which reproduction ceased, using the populations selected for different ages of reproduction described earlier (Rose et al., 2002; Rauser et al., 2006). Further experiments showed that the shift can be detected after only 24 generations of selection for early reproduction (Rose et al., 2002). Since some individuals continue to live during a period of the life span when deleterious mutations are postulated to accumulate (mutation accumulation) or when mutations manifest their negative effects upon survival (antagonistic pleiotropy), it is postulated that alleles must be present that have adult-persistent positive effects upon survival (or effects within a large age window) (Charlesworth, 2001; Rauser et al., 2006). The period with the mortality rate plateau, also called “late life,” has been correlated with a shift in several physiological traits (Shahrestani et al., 2016; Zhang et al., 2016).

When experimental evolution was first applied to the evolution of aging, measurements of life history traits were analyzed using sophisticated mathematical models that allowed the derivation of mortality rates, rates of aging, genetic variances, and correlations between any of the measured variables (Promislow et al., 1996; Pletcher et al., 1999; Stroustrup, 2018). The advent of low-cost high throughput sequencing has allowed a transition from genetic parameters to genotypes. The goal is to link phenotypes to changes at the nucleotide level, an approach called “evolve and resequence” or E&R (Long et al., 2015; Schlötterer et al., 2015). One of the first E&R studies in a sexually reproducing organism looked at *Drosophila melanogaster* populations selected over 600 generations for shortened development times (Burke et al., 2010). Comparisons between the selected and control populations revealed almost 700,000 single nucleotide polymorphisms (SNPs) that differed in frequency between the two conditions, of which approximately 37,000 were non-synonymous changes in coding regions. Statistical analysis identified 662 SNPs of high significance, in 506 genes. Most of the identifiable genes were related to developmental processes. When the heterozygosity of the genome was mapped, the pattern was interpreted as consistent with high levels of standing genetic variation that can be strongly biased toward particular alleles without leading to allele fixation. Some subsequent E&R studies in *Drosophila* have come to similar conclusions with regard to heterozygosity changes, allele frequency divergence during selection, and the probable mechanism of evolutionary change. For example, Graves et al. (2017) sequenced populations selected for 3 different windows of reproduction and consequently 3 life span profiles (Rose et al., 2002; Burke et al., 2016). Heterozygosity was calculated using the allele frequencies of 1 million SNPs. Hundreds of generations of selection lead to significant decreases of heterozygosity at dozens of locations on each chromosome (regions spanning hundreds of kb) but no signatures of allele fixation. There are multiple methodological issues associated with E&R studies in metazoans, such as the number of replicate populations (Kofler and Schlötterer, 2014), the number of individuals in each population over the course the selection protocol (Kofler and Schlötterer, 2014), the duration of the selection in terms of generations (Kofler and Schlötterer, 2014), the number of sequencing reads needed to minimize errors (Futschik and Schlötterer, 2010), the use of pooled samples versus sequences from individuals (Schlötterer et al., 2015), the mapping of the sequences, the presence of haplotypes in the populations (Franssen et al., 2015; Otte et al., 2021), the presence of structural variants such as inversions (Barghi et al., 2017), and the need to use multiple statistical tools based upon population genetic theory (Schlötterer et al., 2015). While the study by Burke et al. (2010) only examined coding region changes, changes in regulatory regions can be inferred from differential gene expression in selected populations relative to controls. This has been done for *Drosophila* populations selected for different temperature regimes (Mallard et al., 2018; Hsu et al., 2021) or different life spans (Remolina et al., 2012; Barter et al., 2019). Differential gene expression also provides a measure of phenotypic divergence in the selected populations. Mallard et al. (2018) sampled a natural population of *Drosophila simulans* prior to selection for high temperature; temperature adaptation had already been shown to be under variable selection in natural populations of *Drosophila simulans* and *melanogaster*. E&R supplemented with transcriptome analysis implicated two haplotypes that segregate at intermediate frequencies in natural populations. Within these haplotypes, transcriptional analysis indicated two genes had large effects in the adaptation to high temperature, an AMPK subunit and regulator. Metabolic changes, as revealed by other gene expression changes and physiological assays, were consistent with AMPK-mediated adaptation to high temperatures.

Work in *Drosophila* species has been central to every issue examined in experimental evolution. A recent publication (Flatt, 2020) comprehensively reviews a literature spanning decades.

### Different Types of Pleiotropy, From an Evolutionary Perspective

Population genetics theory predicts that when there is pleiotropy, or *concurrent pleiotropy* using the terminology adopted earlier, and a mutation or allele is selected, then the pleiotropy will reduce the amount of selection (Stern and Orgogozo, 2008). Experimental evidence from yeast genomic screens indirectly supports this prediction, in that the deletion of genes (an extreme allele change) with a higher degree of pleiotropy lead to a greater reduction in fitness compared to genes with a lower degree of pleiotropy (Cooper et al., 2007). In our treatment secondary effects are separated from the primary selected phenotype, therefore the secondary effects would be regarded as having negative effects upon selection (Figure 1).

*Antagonistic pleiotropy* proposes that a gene may have a positive effect early in life that is under selection and secondarily have a negative effect later in life (Williams, 1957), a byproduct effect (Figure 1). A positive early life effect would mean increased adaptation or reproductive efficacy, leading to a higher number of offspring, while a negative late life effect would mean increased mortality or frailty. In order to make the terminology consistent with the mutation accumulation theory and facilitate a discussion of genetic variation that contributes to aging, antagonistic pleiotropy can be redefined as a mutation that has a positive effect on fitness early in life and a negative effect on fitness late in life (terminology used in Moorad and Promislow, 2008). Since mutations can vary in their pleiotropic effects depending upon where they are located in a gene (cis regulatory elements compared to transcription units, Stern and Orgogozo, 2008), there is a further incentive to focus upon mutations and not genes.

*Positive pleiotropy* can be defined as occurring when a mutation has a positive early life effect that is under selection, and a secondary late life effect (byproduct effect) that is also positive. This differs from previous use of the term *positive pleiotropy* (Kimber and Chippindale, 2013; Maklakov et al., 2015) in that the positive effects are attributed here to the mutation, not the genetic factor (previous usage). Pleiotropy that involves an early life negative effect (*negative pleiotropy*) is assumed by evolutionary theory to be eliminated by natural selection (irrespective of whether the secondary late life result is negative or positive). A case where this assumption may not hold would be small populations subject to genetic drift (see below).

An example of positive pleiotropy would be the *Indy* mutation in *Drosophila* (reviewed in Frankel and Rogina, 2012 and Rogina, 2017). *Indy* mutant flies have both increased egg production when fed a normal diet and increased life spans, indicating positive effects during early and late life (Marden et al., 2003). Evidence for positive pleiotropy has also been found in demographic studies using *Drosophila* (Kimber and Chippindale, 2013).

A small population undergoing genetic drift can acquire a mutation with a small negative effect early in life and a secondary or byproduct effect (positive or negative) later in life. Small negative effects earlier in life would probably escape selection and be effectively neutral in such a population (Stern and Orgogozo, 2009; Lanfear et al., 2014). All pleiotropic effects of the mutation would therefore not be subject to selection, and this quality can be highlighted by calling the late age effects *neutrality-based byproduct effects* (Figure 1). Since one effect occurs earlier and the others later, the earlier effect can be called primary and the later effect secondary. In contrast to this would be the secondary effects of pleiotropies whose primary effect is subject to selection, which we propose to describe as *selection-based byproduct effects* (Figure 1).

There is an additional scenario that could contribute to the evolution of late age phenotypes in both large and small populations. A mutation that has a no phenotype at an earlier age or is neutral with regard to selection could potentially have a negative or positive effect later in life (in order to minimize assumptions, both types of effect are considered). This would also be categorized as a *neutrality-based byproduct effect* (Figure 1). *Neutrality-based byproduct effects* allow chance to have a larger role in creating genetic variability at later ages, and widen the possibilities for mutation accumulation. The role of neutral and slightly negative mutations during evolution has been a consistent topic of theoretical and experimental interest (Kimura and Ota, 1974; Ohta and Gillespie, 1996; Lanfear et al., 2014; Jensen et al., 2019; Wideman et al., 2019).

Work on *Drosophila* aging provides two examples of mutations that could be neutral in early life but be either negative or positive in late life. The microbiota of flies changes drastically with age. A mutation in an innate immune receptor that binds to a late life species of bacteria, inhibiting the effectiveness of that receptor, will be neutral during early life but have a negative effect late in life (Clark et al., 2015). Several fly models of neurodegenerative diseases show that inhibition of enzymes in the kynurenine pathway of tryptophan degradation are protective in older flies, but seem to have no effect in young flies (Breda et al., 2016). Mutations that have a neutral effect at younger ages but a negative/postive effect at later ages are a special case. The mutations in the two examples are adult persistent at the molecular level, but age-specific with late onset phenotypically. Furthermore, the presence of the mutation at an earlier age could be unmasked phenotypically by epistasis.

### Different Types of Pleiotropy, From a Mechanistic Perspective

Studies from molecular and cellular biology have revealed multiple mechanisms underlying pleiotropy. However, moving from genetic or population effects to molecular pathways may require new terminology, so as to explicitly identify this context and the shifted use of “pleiotropy.” Studies of molecular mechanisms are usually idealized for an entire species, and when such studies use genetics, the purpose is to infer how molecules interact based upon the phenotypes in an isogenic or inbred background. The following quote illustrates these characteristics and also shows the great power of this approach when describing the broad phenotypic effects of particular molecules. “Here we show that JNK requires Foxo to extend life span in *Drosophila*. JNK antagonizes IIS, causing nuclear localization of Foxo and inducing its targets, including growth control and stress defense genes. JNK and Foxo also restrict IIS activity systemically by repressing IIS ligand expression in neuroendocrine cells” (Wang et al., 2005). Molecular mechanisms could potentially be divided into *molecule-based* and *network-based pleiotropy*. *Molecule-based pleiotropy* would occur when a particular molecule has multiple effects within a cell or organism. Analysis of a genomic screen of yeast deletion mutants for growth under 21 conditions found that “pleiotropy is generally caused by a single molecular function involved in multiple biological processes” (He and Zhang, 2006), and the degree of pleiotropy was positively correlated with the number of protein-proteins interactions documented for the pleiotropic molecule. Moving from mechanism to evolution, selection for one effect of a pleiotropic molecule might also influence other effects of the molecule. The secondary effects would be expected to occur *concurrently* to the primary selected effect. In order to affect aging, such secondary effects would need to persist into late life. However, the late life effects may not be the same as the early or mid-life effects if the interaction landscape for the pleiotropic molecule shifts.

An example of molecule-based pleiotropy relevant to aging would be a histone deacetylase, which has many target proteins and therefore many effects (Haberland et al., 2009; Frankel et al., 2011). A mutation that affects deacetylase activity and therefore changes a trait associated with one of its targets will also affect traits associated with other targets. The mutation could be subject to selection or genetic drift. *Molecule-based pleiotropy* can therefore lead to either a *selection* or *neutrality byproduct effects*. A mutation affecting the activity of a deacetylase would persist into late life, since most histone deacetylases are necessary for viability.

A molecule that would lead to molecule-based pleiotropy will frequently (but not always) be a regulatory protein. The RPD3 histone deacetylase illustrates how readily such pleiotropy might occur. *Drosophila melanogaster* life span is highly sensitive to the dosage of this protein. Modest decreases in transcript levels, mediated by heterozygosity for null or hypomorphic mutations in the *rpd3* gene, leads to significant increases in life span (Rogina et al., 2002; Frankel et al., 2015). These mutations also lead to changes in metabolism and stress resistance (Woods et al., 2016); some metabolic effects occur in early life, while others occur in late life. RPD3, part of the HDAC I family, is a highly conserved protein (Haberland et al., 2009). Alleles that affect the level of transcription of this gene might readily occur in natural populations of multiple species.

An example of *molecule-based pleiotropy* that does not involve a regulatory protein would be a mutation that affects the levels of a metabolite shared by several different pathways, such as citrate (Frankel and Rogina, 2012). INDY is a member of a family of plasma membrane Na-carboxylate cotransporters. First studied in *Drosophila*, homologs of INDY have subsequently been studied in *C. elegans* and mice (Frankel and Rogina, 2012; Rogina, 2017). INDY has a significant affect upon cytoplasmic levels of citrate. Increased citrate levels in the cytoplasm increases fatty acid synthesis and inhibits glycolysis. Cytoplasmic citrate is linked to mitochondrial citrate via malate transport. Mitochondrial citrate levels affect the rate of respiration. Decreased INDY levels in fly and mouse tissues leads to multiple metabolic changes and an increased number of mitochondria, changes consistent with decreased cytoplasmic citrate levels.

Cells have many networks or pathways, such as signaling networks or metabolic networks. Since networks are usually interconnected, a mutation affecting one network might ramify and affect other networks (Soltow et al., 2010; Riera et al., 2016). This could be called *network-based pleiotropy*. The TORC1 signaling network can receive direct input concerning the abundance of amino acids, and modulate protein synthesis and degradation (Saxton and Sabatini, 2017). The same signaling network can receive input in the form of crosstalk from other signaling networks, such as the insulin and Wnt signaling networks (Evans et al., 2011). A mutation that would affect one network, such as insulin signaling, can secondarily affect another network, such as the TORC1 signaling. Such a mutation could be located in a gene encoding a protein involved in cross-talk between the two networks. Network-based pleiotropy would be likely to affect late life, and many examples of single mutations that extend life span affect such networks (Evans et al., 2011; Riera et al., 2016; Campisi et al., 2019). Network-based pleiotropy could be either positive or negative for late life. What would distinguish such mutations from molecule-based pleiotropy would be their genetic/phenotypic complexity. Given the multiple inputs and outputs for each network, and the multiple gene products contributing to each network, phenotypic effects would be expected to have a high degree of pleiotropy and be dependent upon genetic background and environmental factors. While mechanistic studies try to control for all of these complications, evolutionary scenarios would need to take them into account. Genomic studies of networks aim to unravel much of this genetic and phenotypic complexity by comprehensively mutating and mapping the phenotypes of most of the genes in model organisms (Tyler et al., 2009; reviewed for *C. elegans* in Gunsalus and Rhrissorrakrai, 2011).

### Aging and Epistasis

In conventional genetics research, epistasis describes the interaction of two or more genes to produce a phenotype not present with each gene by itself, an absence of additivity for similar phenotypes in multiple genes, or a blockage of one gene’s phenotype by alleles of a second gene (Brodie, 2000; Phillips, 2008). Epistasis has a distinct but overlapping meaning in population genetics. Attempts to link epistasis to molecular mechanisms and developmental pathways can lead to further shifts in meaning (Brodie, 2000; Phillips, 2008). The molecular mechanisms underlying epistatic gene interactions include gene products that are in the same pathway or the same network, and gene products that form protein complexes. However, a set of loci can exhibit epistasis by many possible mechanisms involving proteins, RNA molecules, and DNA sequences (Moore and Williams, 2005). Phillips (2008) suggests the terms *compositional* and *statistical epistasis* to distinguish the usages in conventional and population genetics respectively, and his terminology will be used in the following discussion as well.

In the context of age-structured phenotypes, compositional epistasis could either be persistent throughout adult life or age-specific. An adult persistent phenotype exhibiting epistasis would potentially be unchanging, increasing, or decreasing with age. Age-specific effects of most relevance to intrinsic mortality would either have the epistasis begin or end at later ages. Age specificity could occur by the addition or loss of interacting gene products at advanced ages. Alternatively, if there is an accumulation of late-acting mutations, some of these may have epistatic effects. Adult persistent phenotypes could gain age-specificity if there is a quantitative change with age that brings the epistasis phenotype above or below a critical threshold. Adult persistent epistatic phenotypes could potentially be subject to selection. Quantifying that selection, however, relies upon a shift to statistical epistasis. There is substantial debate concerning how to model the role of statistical epistasis in selection (Brodie, 2000; Phillips, 2008; Payne and Wagner, 2019).

### Transcriptional Changes During Aging

A number of studies have attempted to understand the aging phenomenon in terms of age-dependent transcriptional changes. Candidate gene studies have examined changes in the expression of a particular gene with age, and correlated this with the effects of under or overexpression of the gene on mortality or age-related phenotypes. This approach has been useful for identifying particular regulatory molecules, signaling networks, and metabolic networks affecting longevity (Gems and Partridge, 2013). An alternative approach has examined transcriptome changes with age, with the aim of identifying large scale molecular changes underlying the tissue and organismic changes associated with aging. The transcriptome can also potentially provide data supporting one of the evolutionary theories of aging. The following summary applies to microarray and bulk RNA sequencing studies (reviewed by Stegeman and Weake, 2017; Perez-Gomez et al., 2020). (1) Only a low percentage of the total transcriptome changes with age, usually 3–4% of the expressed genes in most tissues for most species. (2) While many differentially expressed genes can be categorized by gene ontology (GO), there is often a large group of unknown function. (3) Genes related to immune and stress responses are usually up-regulated, and genes related to oxidative phosphorylation are usually down-regulated. (4) The actual genes that change their expression with age appear to be different in each species, even when there are GO similarities. A study examining 17 mouse tissues using bulk RNA sequencing (Schaum et al., 2020) reported that most genes whose expression changes with age are tissue specific and are concentrated in liver, kidney, and adipose tissue. Single-cell RNA sequencing in mice has provided a finer focus (Tabula Muris consortium, Almanzar et al., 2020). The mixture of cell types in all tissues change with age, but each cell type was stable with age (with the exception of immune cells). The same group compared age-related gene expression changes detected by bulk RNA sequencing to the changes detected by single-cell RNA sequencing. Changes detected by bulk RNA sequencing were due to both altered numbers of cell types in a tissue and changes within particular cell types. A different study examined mouse brain tissue using single-cell RNA sequencing and showed preservation of both cell identity and cell type composition with age (Ximerakis et al., 2019). Similar results were obtained with single cell RNA-sequencing of *Drosophila* brain (Davie et al., 2018).

Transcriptome analysis during aging has also been performed in the context of experimental evolution (Barter et al., 2019). Populations of *Drosophila melanogaster* were selected for early and mid-life reproduction, with mean longevities of 25 and 41 days (males and females averaged together, from Rose et al., 2002). For each selection regime, one set of five replicates was selected for almost 1,200 generations and another set for 327 generations (two selection regimes, within each regime two generational spans). Gene expression profiles aligned for different replicates, and also aligned for similar selection regimes. Out of 3,994 genes (protein coding and non-coding RNA) expressed at significant levels, 906 showed differential expression when comparing early and mid-life selection regimes. The two time points used for these comparisons occurred when the early populations are aging but the mid-life populations are not, based upon mortality rates. The differentially expressed protein-coding genes revealed no gene ontology patterns and almost no overlap with published lists of *Drosophila* genes that change their expression with age (GenAge database, part of the Human Aging Genomic Resources project; Tacutu et al., 2018).

It has been postulated that the decay of gene regulation is one of the factors underlying the aging pattern (Lai et al., 2019). The relevant data from metazoan model systems is complex. Early studies in *Drosophila*, using beta-galactosidase enhancer trap lines, showed that gene regulation was maintained throughout adult life span (Helfand et al., 1995; Rogina and Helfand, 1996, 1997; Rogina et al., 1997, 1998). Some genes showed an unchanging expression level, others showed a steady decrease, and others showed a steady increase. In addition, a few genes showed a more complex pattern that was reproducible and stereotyped throughout the life span, as shown by modulations of longevity (Rogina and Helfand, 1996, 1997). Variation in expression of the same gene, using cohorts of age-matched flies (usually 10), did not change with age (Rogina et al., 1998). Single-cell RNA sequencing of mouse brain (Ximerakis et al., 2019) measured the variance for all genes in 11 cell types, and found on average no change with age. RNA from single *Drosophila* hearts was used to assay the expression of three genes by qPCR, and while there was great variability, this variability did not change with age (Cannon et al., 2017). Bulk RNA sequencing of four rat tissues looked at the standard deviation for age-regulated genes and only saw an increase at the latest age for two tissues, no change in one tissue, and a clear change in one tissue (Shavlakadze et al., 2019). Isolated mouse cardiomyocytes were examined by RT-PCR for 12 nuclear genes, and the transcription of all 12 showed increased cell-to-cell variation with age (Bahar et al., 2006). Bulk RNA sequencing of individual mouse livers showed an increased variance in old livers, though the sample size was only three young and three old livers (White et al., 2015). Single-cell RNA sequencing of stimulated mouse CD4 + T cells found a higher variability in cells from older mice (Martinez-Jimenez et al., 2017). In the Tabula Muris study, the expression profiles of all cell types were stable with age with the exception of immune cells. It is possible that immune cells may be particularly susceptible to age-related damage. Overall, the data does not yet support the view that there is a global dysregulation of transcription with age.

### Dietary or Caloric Restriction

Decreased nutrition (dietary or caloric restriction, DR) has been found to both increase life span and slow physiological aging in a phylogenetically diverse set of organisms (Weindruch and Walford, 1988; Mair and Dillin, 2008; Piper et al., 2011), though a few non-model organisms fail to show this effect (Nakagawa et al., 2012). The DR response was first demonstrated in rodents, where the effects are highly sensitive to genetic background (Swindell, 2012; Liao et al., 2013; Ingram and de Cabo, 2017). The DR response can be readily induced in *Drosophila* (Bross et al., 2005), where the effect is also sensitive to genetic background (Grandison et al., 2009; Wilson et al., 2020). *Drosophila* has been used to measure longevity in response to a wide range of food formulations, confirming that restriction of particular nutrients can increase longevity and it is not solely a calorie effect (Skorupa et al., 2008; Tatar, 2011; Sohal and Forster, 2014; Tatar et al., 2014). DR can be induced in *C. elegans*, but it is complicated since the usual food source is bacteria, so a reduction of calories concurrent with a maintenance of essential nutrients is not possible (Lee et al., 2006; Houthoofd et al., 2005, 2007). In *S. cerevisae*, DR effects are strain specific and need to be defined in the context of a unicellular organism that quickly adjusts to different energy sources (Schleit et al., 2012). DR leads to a marked decrease in reproduction, at least in *Drosophila* and mammals, often reversible upon calorie or nutrient increase. From an evolutionary perspective, it has been proposed that DR can be a diapause-like program that delays reproduction and extends adult life in response to adverse environmental conditions (Kirkwood and Austad, 2000; Tatar and Yin, 2001). DR can either be the result of divergent or convergent evolution. In the former case there would be phylogenetically conserved upstream regulators. Several candidates for upstream regulators have been proposed, but interspecies differences have made the evolutionary pattern ambiguous (Mair and Dillin, 2008). The multiplicity of distal or downstream DR effects is consistent with either convergent of divergent evolution. A recent study examined intra-specific variation for DR-mediated changes in life span, using 161 genetically diverged inbred strains of *Drosophila* (Wilson et al., 2020). Under normal feeding there was great variation in median life span, DR increased life span for many (83%) but not all strains, and the magnitude of the DR-mediated increase was highly variable. A study using 41 inbred strains of mice obtained a DR-mediated increase in life span for only 5% of the strains in males and 21% of the strains in females (Liao et al., 2010). These results argue for more studies on the interspecific and intraspecific variation of DR-mediated life span effects.

DR appears to have coordinated effects upon mortality and physiological aging in worms, flies, and rodents (Speakman and Mitchell, 2011; Kapahi et al., 2017). The previously mentioned study of different *Drosophila* strains (Wilson et al., 2020) also measured a biomarker of physiological aging, the age-dependent decline in climbing ability. There was great variation in this biomarker between strains, and DR delayed the decline in many (69%) but not all strains. The panel of strains showed no correlation between the effect of DR upon life span and the biomarker. This is the first evidence suggesting that DR may induce genetically distinct responses, and furthermore, that these responses may not be conserved across genotypes.

The sensitivity of the DR response to genetic background supports the view that it is a program superimposed upon a complex phenomenon arising from the alleles of a large number of genes. Further support for this view is provided by a study of rat aging using single-cell RNA-sequencing (Ma et al., 2020). Aging affected the expression of a different set of genes in each cell type and tissue, DR reversed many but not all of these age-dependent changes, and the effects of DR were also cell type and tissue specific.

### Aging as an Emergent Phenomenon

Evolutionary theory allows for various types of byproduct effects that can affect late life, both negatively and positively. If pleiotropy is viewed mechanistically, molecule-based and network-based pleiotropy make late life effects likely. Research on model systems has already shown that there are a large number of mechanisms by which single mutations can affect late life, supporting the possibility that populations accumulate diverse positive and negative late life effects. It is likely that late life is subject to little selection due to rapidly decreasing population size and lack of late life reproduction in most species, making it unlikely that aging is under simple regulatory control (Moorad et al., 2019). Instead, it could potentially be an emergent property of the many byproduct effects that affect late life (both selection-based and neutrality-based) and the accumulation of mutations primarily affecting late life. Each species will have its own constellation of byproduct effects and late acting mutations. This will translate into a large and complex mixture of genetic variation that will distribute across the individuals in populations. Some of the mutations affecting aging may be shared between species, due to conservation of molecules or networks, while others will be species specific (Bauer et al., 2010). Characteristic lifespans for different species would be another emergent property. Superimposed upon this pattern of aging would be physiological responses to environmental insults common to aging animals, such as stress and infection. Such responses could contribute to the aging pattern. These responses would also consist of both conserved and species-specific components. While it is unclear how age-related tissue dysfunction connects to organism mortality, tissue specific changes with age would be expected to contribute to the species-specific pattern.

### Species-Specific Aging

The existence of species-specific aging is well established (Finch, 1990; Promislow, 1991; Jones et al., 2014). Most species that age have a characteristic intrinsic mortality rate, correlating with the rate of aging and maximum life span. There is great diversity in the patterns of intrinsic mortality and fertility across life span, using data from a broad phylogenetic cross-section of species (Nussey et al., 2013; Jones et al., 2014; Johnson et al., 2019). This includes species that do not age, ranging from isolated examples to entire clades. The common denominator is species specificity for mortality and fertility rates. This confirms earlier studies that compared the life spans of closely related *Drosophila* species (Schnebel and Grossfield, 1983; Wit et al., 2015). One hypothesis for the evolution of species-specific life span postulates an inverse relationship between extrinsic mortality and life span (Kirkwood and Austad, 2000). However, the current data on patterns of intrinsic mortality show multiple exceptions to the postulated inverse relationship. There are also theoretical considerations that may limit the applicability of this inverse relationship (Moorad et al., 2019). Since the study by Jones et al. (2014), an open-source database has been established as a repository for demographic data pertaining to animal and plant species (Salguero-Gómez et al., 2016). Currently there are 415 animal and 758 plant species, representing a broad cross-section of clades (Healy et al., 2019). This rapidly expanding life history data should greatly aid studies on the evolution of aging.

### Germline Versus Somatic Mutations

Evolutionary theory usually assumes that the mutations driving changes in traits are germline mutations, since the focus is on population changes over multiple generations. However, somatic mutations might also be relevant to a late life phenomenon like aging. One theory of aging proposes a causal link between aging and the accumulation of somatic mutations (Zhang and Vijg, 2018; Schumacher et al., 2021; Vijg, 2021). Arguing against this link would be a pattern of aging that is species-specific. If aging is closely related to intrinsic mortality rates, a central principle in theories of aging, and if a species-specific pattern of aging was caused by somatic mutations, there would have to be species-specific constraints on the types or quantity of somatic mutations that occur. Such constraints would be related to DNA repair mechanisms. Data to support patterns of somatic mutations remains very difficult to obtain (Martincorena and Campbell, 2015; Zhang and Vijg, 2018; Schumacher et al., 2021). An alternative approach is to compare the life spans of closely related species. Within the genus *Drosophila* there is considerable variability in life spans (Schnebel and Grossfield, 1983; Wit et al., 2015). There is also widespread variation in longevity within the same species, as has been documented with divergent inbred *Drosophila* and mouse lines (Gelman et al., 1988; Liao et al., 2010; Wilson et al., 2020). A complex trait such as DNA repair, dependent upon dozens of enzymes linked to multiple networks, would not be expected to vary widely in closely related species or within populations of the same species.

The issue of somatic and germline mutations also arises in discussions of cancer, which is often also correlated with late life. Underlying the general pattern of cancer progression is a bewildering variety of molecular differences. Aging may also involve a great deal of molecular heterogeneity underneath gross physiological similarities. However, one major difference between cancer and aging is that cancer is a disease of replicating cells, whereas aging is a condition characterized by the cessation of cell division - it often involves either senescent cells, stem cells that have reduced replication potential, or post-mitotic differentiated cells. The replication of pre-cancerous cells can lead to a form of evolution in miniature, with selection for faster cell division and metastatic phenotypes (Martincorena and Campbell, 2015; Turajlic and Swanton, 2016; Wu et al., 2016). Such a process would not be expected to occur in aging cells. In the absence of evidence for species-specific constraints on somatic mutations, it might be most reasonable to assume that aging is mainly affected by germline mutations.

### Cumulative Damage Theory of Aging

Other theories of aging do not invoke mutations, but instead propose the accumulation of various forms of molecular and organelle damage (Gems and Partridge, 2013; Vijg and Suh, 2013; Gorbunova and Seluanov, 2016). This presupposes that particular forms of damage overwhelm repair or degradation (proteolysis, autophagy) mechanisms over time. Some genetic studies have enhanced particular mechanisms of repair or degradation, and have shown longevity extension (Lapierre et al., 2013; Pyo et al., 2013). Other studies have enhanced mechanisms that remove free radicals, a metabolic byproduct that is a particularly potent agent of damage, leading to increased longevity (Melov et al., 2000; Schriner et al., 2005). Subsequent work has cast doubt on the results with enhanced antioxidant mechanisms (Gems and Partridge, 2013; Edrey and Salmon, 2014). Experimental support for a link between aging and the accumulation of other (non-oxidative) types of damage is still incomplete (Gems and Partridge, 2013; Vijg and Suh, 2013; Kong et al., 2014; Zhang and Vijg, 2018). Nevertheless, if cumulative damage was a factor in aging, it would be consistent with the accumulation of pleiotropic mutations and byproduct effects. Such mutations could contribute to species or clade-specific capacities for repair, degradation, or removal of damage agents, and therefore contribute to species or clade-specific aging patterns and mortality rates. Repair and degradation capacities may also be subject to selection of various kinds, leading to potential enhancements or decreases suited to the life history of the species. The disposable soma theory proposes that repair and degradation have costs, and those costs significantly impinge upon the metabolic limits of an organism (Maklakov and Immler, 2016). Longer lived species would invest more resources into repair and somatic maintenance, while shorter lived species would optimize for reproduction and germline integrity (Kirkwood, 2002). There is some evidence consistent with this theory (reviewed in Maklakov and Immler, 2016) and some evidence that is not consistent with the theory from wild populations (reviewed in Johnson et al., 2019) and laboratory selection experiments (Chen and Maklakov, 2012; Wit et al., 2013; Zajitschek et al., 2019).

Mechanistic studies of aging highlight changes over the life span that result in an accumulation of physiological and molecular deterioration. An evolutionary perspective provides a potential complication for this point of view (Rose et al., 2012). The mortality rate plateau, which seems to be accompanied by a plateau of at least some types of physiological decline in a model species (Shahrestani et al., 2016), highlights adaptation and selection as part of the aging phenomenon. Throughout most of the reproductive period of adulthood the force of natural selection upon fitness declines, leading potentially to lessened adaptation, which in turn will lead to physiological declines. However, at a particular point some or most of these declines flatten, allowing some fortunate individuals to live extended life spans. Superimposed upon this will be robust physiological responses to stresses and insults, further complicating a trajectory of decline. This push and pull may be most pitched in the immune system, which in *Drosophila* has been shown to respond to changes in bacterial loads across the life span (Clark and Walker, 2018), while immune capacity itself may be declining (Fabian et al., 2018; Garschall and Flatt, 2018).

## Summary

Over the last several decades molecular genetic studies have provided a deep understanding of mechanisms operating at the cellular, tissue, and organismal level, including mechanisms relevant for aging. From a therapeutic point of view, understanding aging as it occurs in humans is of vital importance for the maintenance of health, the treatment of disease, and the containment of a looming challenge for health care systems (López-Otín et al., 2013; Kennedy et al., 2014; Campisi et al., 2019). Aging appears to be more prominent in some branches of the phylogenetic tree and less prominent in others (Jones et al., 2014). From an evolutionary perspective, accounting for aging has proven to be highly productive for the development of evolutionary theory (Flatt and Partridge, 2018; population genetic models of aging are discussed in Moorad and Promislow, 2008 and Moorad et al., 2019). Incorporating mechanistic understandings into the evolutionary view of aging may be aided by highlighting the roles of pleiotropy, epistasis, and secondary mutation effects (byproduct effects).

Pleiotropy was originally a genetics concept (Caspari, 1952: Stearns, 2010), but it has since been applied to a wider range of contexts. Pleiotropy as a genetic phenomenon can be subdivided into two age relevant types, concurrent or sequential, depending upon whether the multiple effects occur in the same life history stage or in different stages. The concept can be extended to include byproduct effects accumulating without selection. We have defined molecule-based and network-based pleiotropy, in order to highlight and distinguish mechanistic approaches to pleiotropy. These new terms will hopefully have particular relevance for aging, where discussions toggle between mechanisms and evolutionary issues.

One of the most prominent theories accounting for aging and age-dependent mortality rates postulates cumulative damage leading to stochastic failure of tissues and the organism. Specific mechanisms included in this theory are oxidative damage or somatic mutation. The disposable soma model states that repair capacities are limited by evolutionary constraints, leading to this cumulative damage. At the present time these theories cannot account for the full range of aging patterns in all species. Perhaps aging can be viewed as the net result of hundreds of byproduct effects combined with the accumulation of late-acting mutations, encompassing both positive and negative effects upon mortality and vigor. Aging is correlated with a large number of species, tissue, and cell type specific changes at the molecular level. It is possible that aging is an emergent property of hundreds of effects, some conserved, some fixed at the species level, and some that are variable at the population level. According to this view, it would only be a modest exaggeration to say aging is all trees and no forest.

## Author Contributions

SF conceived the work and took a lead role in the writing of each draft. BR shaped each iteration and refined each draft. Discussions between SF and BR over the course of a 25-year collaboration provided the foundation for this work. Both authors contributed to the article and approved the submitted version.

## Conflict of Interest

The authors declare that the research was conducted in the absence of any commercial or financial relationships that could be construed as a potential conflict of interest.

## Publisher’s Note

All claims expressed in this article are solely those of the authors and do not necessarily represent those of their affiliated organizations, or those of the publisher, the editors and the reviewers. Any product that may be evaluated in this article, or claim that may be made by its manufacturer, is not guaranteed or endorsed by the publisher.
